# Ultrasound-Guided Biceps-Tracking Access to the Superior Labral-Biceps Anchor Complex: A Technical Report With Cadaveric Demonstration of Gross Injectate Localization

**DOI:** 10.7759/cureus.111637

**Published:** 2026-06-28

**Authors:** Sang-Hyun Kim, U-Young Lee, Yonghyun Yoon, Jihyo Hwang, Seungbeom Kim, Youngmo Kim, Teinny Suryadi, Anwar Suhaimi, King Hei Stanley Lam

**Affiliations:** 1 Anatomy, College of Korean Medicine, Woosuk University, Jeonbuk-do, KOR; 2 Anatomy, Catholic Institute for Applied Anatomy, College of Medicine, Catholic University of Korea, Seoul, KOR; 3 Orthopedics, International Academy of Musculoskeletal Medicine, Hong Kong, HKG; 4 Orthopedics, International Academy of Regenerative Medicine, Incheon, KOR; 5 Orthopedics, Musculoskeletal Ultrasound (MSKUS), San Diego, USA; 6 Orthopedic Surgery, Hallym University Kangnam Sacred Heart Hospital, Seoul, KOR; 7 Orthopedic Surgery, Incheon Terminal Orthopedic Surgery Clinic, Incheon, KOR; 8 Pain Medicine, Miso Pain Clinic, Suwon, KOR; 9 Orthopedics, Incheon Terminal Orthopedic Surgery Clinic, Incheon, KOR; 10 Physical Medicine and Rehabilitation, Medistra Hospital, Jakarta, IDN; 11 Physical Medicine and Rehabilitation, Synergy Clinic, Jakarta, IDN; 12 Physical Medicine and Rehabilitation, Hermina Podomoro Hospital, Jakarta, IDN; 13 Rehabilitation Medicine, University Malaya Medical Centre, Kuala Lumpur, MYS; 14 Rehabilitation Medicine, University Malaya, Kuala Lumpur, MYS; 15 Clinical Research, International Academy of Regenerative Medicine, Incheon, KOR; 16 Clinical Research, The Indonesian Malaysian Regenerative Institute, Jakarta, IDN; 17 Clinical Research, The International Association of Musculoskeletal Medicine, Kowloon, HKG; 18 Faculty of Medicine, The Chinese University of Hong Kong, New Territories, HKG; 19 Faculty of Medicine, The University of Hong Kong, Hong Kong, HKG; 20 Clinical Research, The Hong Kong Institute of Musculoskeletal Medicine, Kowloon, HKG

**Keywords:** biceps anchor, cadaveric validation, procedural technique, shoulder instability, shoulder pain, shoulder ultrasound, superior labrum, superior labrum anterior to posterior (slap) lesion, supraglenoid tubercle, ultrasound-guided injection

## Abstract

The superior labral-biceps anchor complex is a technically difficult ultrasound target because of its deep location, close relationship to the glenohumeral joint, and partial obscuration by adjacent osseous structures. In current practice, image-guided injections for suspected superior labral pathology are generally performed as glenohumeral intra-articular injections rather than as footprint-directed procedures. This technical report describes a structured ultrasound-guided biceps-tracking approach to the superior labral-biceps anchor complex and provides a cadaveric demonstration of gross injectate localization.

A single fresh-frozen male cadaver was studied bilaterally. The long head of the biceps tendon was identified in short axis within the bicipital groove and then sequentially tracked proximally through the rotator interval to its intra-articular origin. Under real-time ultrasound guidance, a 23-gauge, 6-cm needle was advanced using an out-of-plane lateral-to-medial trajectory, and 1 mL of red-stained high-viscosity filler was injected at the intended target in each shoulder. Layer-by-layer dissection was performed approximately one hour later to assess gross filler localization.

In both shoulders, dissection demonstrated focal filler localization at the supraglenoid tubercle/biceps anchor region, with gross injectate localization adjacent to the intended footprint on direct inspection. No diffuse red-filler pooling was observed within the glenohumeral joint cavity, and no gross posterior, medial, or anterior extension beyond the supraglenoid tubercle was identified. A consistent technical limitation was the narrow acoustic and spatial window between the coracoid process and clavicle. Interpretation of these findings should be cautious because localization was assessed by gross anatomical evaluation only, and the injectate was a high-viscosity surrogate material whose dispersion characteristics may differ from those of clinically used injectates.

These findings suggest preliminary anatomical feasibility of ultrasound-guided footprint-level access to the superior labral-biceps anchor complex in this cadaveric specimen. Because this report is based on a single cadaver with bilateral non-independent observations, gross assessment only, and a non-clinical surrogate injectate, the findings should be interpreted as descriptive rather than as evidence of reproducibility, safety, procedural accuracy, or clinical efficacy.

## Introduction

The glenohumeral joint is inherently predisposed to instability because of the limited osseous constraint between the humeral head and glenoid. Consequently, shoulder stability depends heavily on soft-tissue structures, including the glenoid labrum, capsuloligamentous complex, rotator cuff, and surrounding musculature [1,2]. The glenoid labrum is a fibrocartilaginous structure that deepens the glenoid concavity, contributes to negative intra-articular pressure, and serves as an important attachment site for the glenohumeral ligaments and the long head of the biceps tendon (LHBT) [3]. Pathology involving the superior labrum near the biceps anchor, classically described as superior labrum anterior to posterior (SLAP) lesions, is a recognized source of shoulder pain and dysfunction [4,5].

Diagnosis and management of superior labral pathology remain challenging. Physical examination maneuvers developed specifically for SLAP lesions have shown variable and often limited standalone diagnostic performance. Systematic review evidence demonstrates substantial heterogeneity in reported sensitivity and specificity across studies, and no single maneuver consistently demonstrates reliable diagnostic accuracy in isolation [6,7]. Magnetic resonance imaging and magnetic resonance arthrography can improve diagnostic confidence in suspected superior labral pathology, but interpretation may still be complicated by age-related changes, concomitant intra-articular abnormalities, and interobserver variability. Musculoskeletal ultrasonography is widely used for real-time shoulder assessment and image-guided intervention; however, the superior labrum and biceps anchor remain difficult ultrasound targets because of their depth, proximity to the joint space, and partial obscuration by adjacent bony structures [8,9]. Recent evidence reviews and consensus statements have further emphasized that superior labrum-biceps complex pathology should be interpreted within a broader clinical framework that includes pathoanatomy, patient factors, imaging findings, and associated shoulder disorders [10,11].

Management is similarly heterogeneous and depends on the patient's age, activity level, symptom profile, tissue quality, tear pattern, and associated pathology [10-15]. Even in active patients, the relative roles of SLAP repair and biceps tenodesis remain debated [13-15]. Biologically oriented strategies, including platelet-rich plasma, have also been explored in selected labral repair settings, but evidence supporting non-surgical biologic injection for isolated SLAP pathology remains limited [16]. Before any therapeutic hypothesis can be tested, however, the more basic methodological question is whether ultrasound guidance can provide anatomical access to the superior labral-biceps anchor footprint itself.

In clinical practice, image-guided interventions for suspected superior labral pathology are generally performed as glenohumeral intra-articular injections under fluoroscopic or ultrasound guidance rather than as procedures specifically intended to localize injectate at the superior labral footprint [17-23]. This distinction may be methodologically relevant in future studies involving biologically active, scaffold-associated, or depot-forming formulations if local retention at the labrum-enthesis interface is considered important. Recent cadaveric work has also shown that injectate distribution within the shoulder may vary substantially depending on the selected ultrasound-guided approach, supporting the broader concept that joint access and target-region distribution are not necessarily identical anatomical events [24].

The superior labral-biceps anchor complex presents a distinct technical challenge because the target is deep, partially obscured by adjacent osseous structures, and best conceptualized in continuity with the proximal course of the LHBT [25-28]. The rotator interval, a triangular space between the supraspinatus and subscapularis through which the LHBT courses proximally, provides an intermediate sonographic corridor during this tracking process [27]. Rather than attempting to target the superior labral region directly from the outset, a structured biceps-tracking strategy follows the LHBT proximally from the bicipital groove to its attachment at the supraglenoid tubercle/superior labral region. In plain terms, the tendon is used as an anatomical guide to the biceps anchor. The aim of this technical report was to describe an ultrasound-guided biceps-tracking approach to the superior labral-biceps anchor complex and to assess gross injectate localization in a fresh-frozen cadaveric specimen.

## Technical report

Study design and ethical considerations

This was a proof-of-concept cadaveric technical report designed to describe ultrasound-guided access to the superior labral-biceps anchor complex and to document gross injectate localization at the intended anatomical target. Because this was a single-donor descriptive cadaveric report with bilateral non-independent observations, inferential statistical analysis was not appropriate.

The cadaveric component was reviewed by the Institutional Review Board of the Catholic University of Korea and was exempted from full ethical review because it involved cadaveric specimens only and no identifiable personal data (IRB No. MIRB-면20260120-006; approved January 20, 2026). Representative ultrasound tracking images and videos were obtained from a healthy volunteer after written informed consent for acquisition and publication.

Cadaveric specimen and positioning

A single fresh-frozen male cadaver (age: 69 years, height: 166 cm, and weight: 57 kg) was used. The specimen had been stored at -20 °C and was thawed at 4°C for 48 hours before the procedure. Complete thawing was confirmed by palpation before injection.

Both shoulders were studied using the same standardized protocol. Because both shoulders originated from the same donor, the two observations were not considered statistically independent and are reported descriptively as separate procedural attempts. Gross inspection during dissection did not reveal marked structural abnormalities, such as a full-thickness rotator cuff tear or severe degenerative change that would have precluded a standardized approach; however, occult structural pathology or age-related intra-articular changes could not be excluded because no preprocedural imaging or histologic confirmation was performed.

For the procedure, the cadaver was placed supine with the shoulder in 0° of abduction and 0° of forward elevation. The elbow was maintained in full extension, and the shoulder was externally rotated to approximately 90° to optimize anterior exposure of the LHBT and rotator interval and to improve the sonographic corridor toward the biceps anchor. A bolster was placed beneath the scapular region to gently distract the humeral head from the glenoid and improve sonographic access to the superior labral region. The reproducibility and tolerability of this position in living symptomatic patients remain uncertain and should be evaluated in future studies.

Ultrasound equipment

Ultrasonographic evaluation was performed using an Alpinion XC90 Elite ultrasound system (ALPINION MEDICAL SYSTEMS Co., Ltd., Seoul, Republic of Korea) with a linear transducer (center frequency: 7 MHz and operating range: 3-19 MHz). Imaging depth was set to 4 cm and dynamic range to 60 dB. Coupling gel was applied to optimize acoustic transmission. All ultrasound examinations and injections were performed by a single orthopedic surgeon (Y.Y.) with more than 10 years of musculoskeletal ultrasonography experience.

Structured biceps-tracking protocol

**Figure 1:**
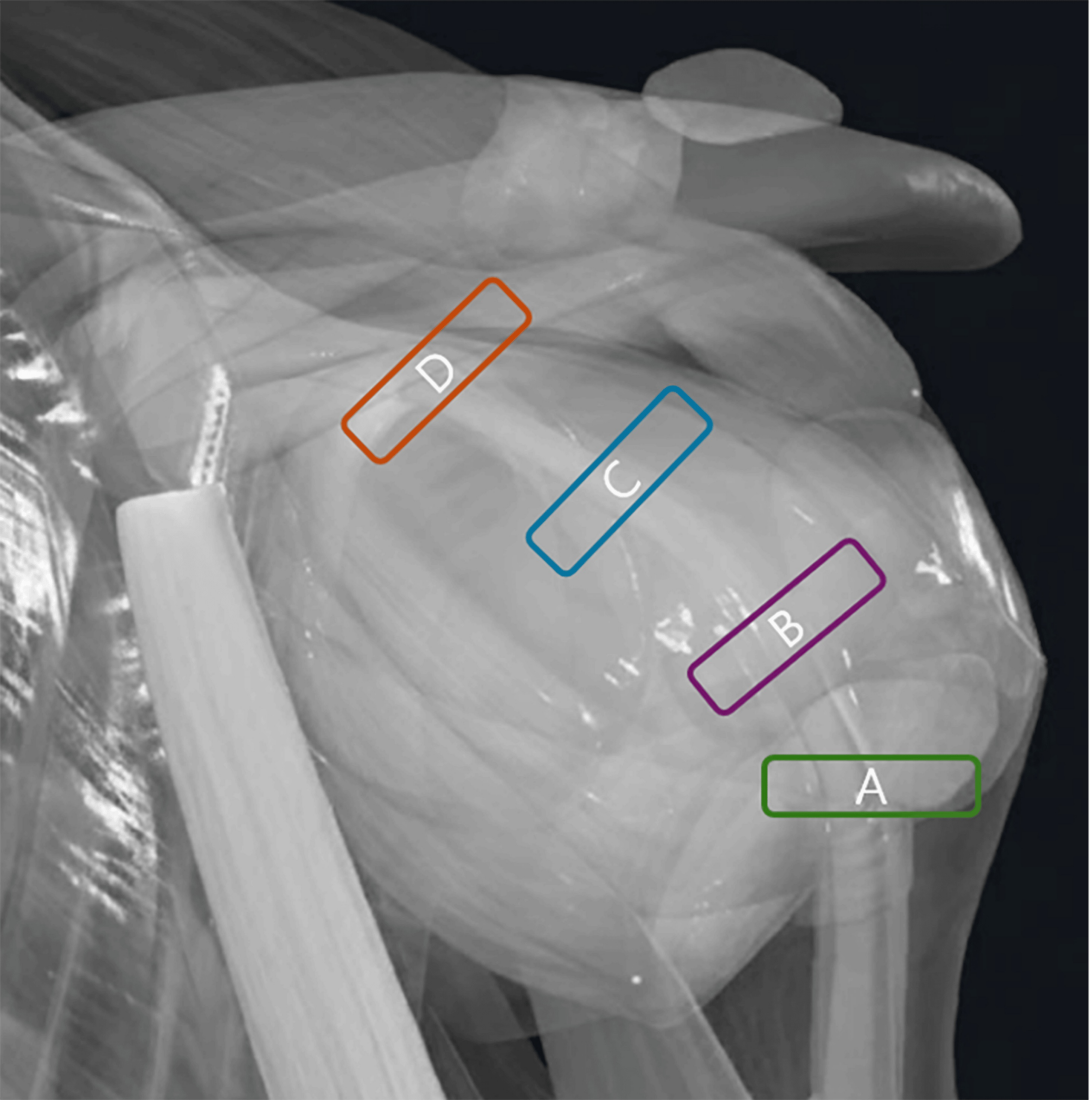
Schematic drawing of standardized probe positions for stepwise short-axis ultrasound tracking to the superior labral-biceps anchor complex Representative static short-axis ultrasound images of the structured biceps-tracking sequence. (A) The long head of the biceps tendon (LHBT) is identified within the bicipital groove. (B) Proximal tracking through the rotator interval region. (C) Visualization of the LHBT entering its intra-articular course. (D) Final confirmation of the biceps anchor at the supraglenoid tubercle/superior labral attachment Image courtesy of Dr. Yonghyun Yoon

The LHBT was first identified within the bicipital groove in short-axis view (Figure 1A). From this reproducible superficial landmark, the transducer was translated proximally while maintaining a consistent short-axis orientation (Figure 1B). The tendon was then sequentially followed through the rotator interval and into its intra-articular course (Figure 1C). Final target confirmation was obtained at the superior labrum-biceps anchor region near the supraglenoid tubercle (Figure 1D).

**Figure 2:**
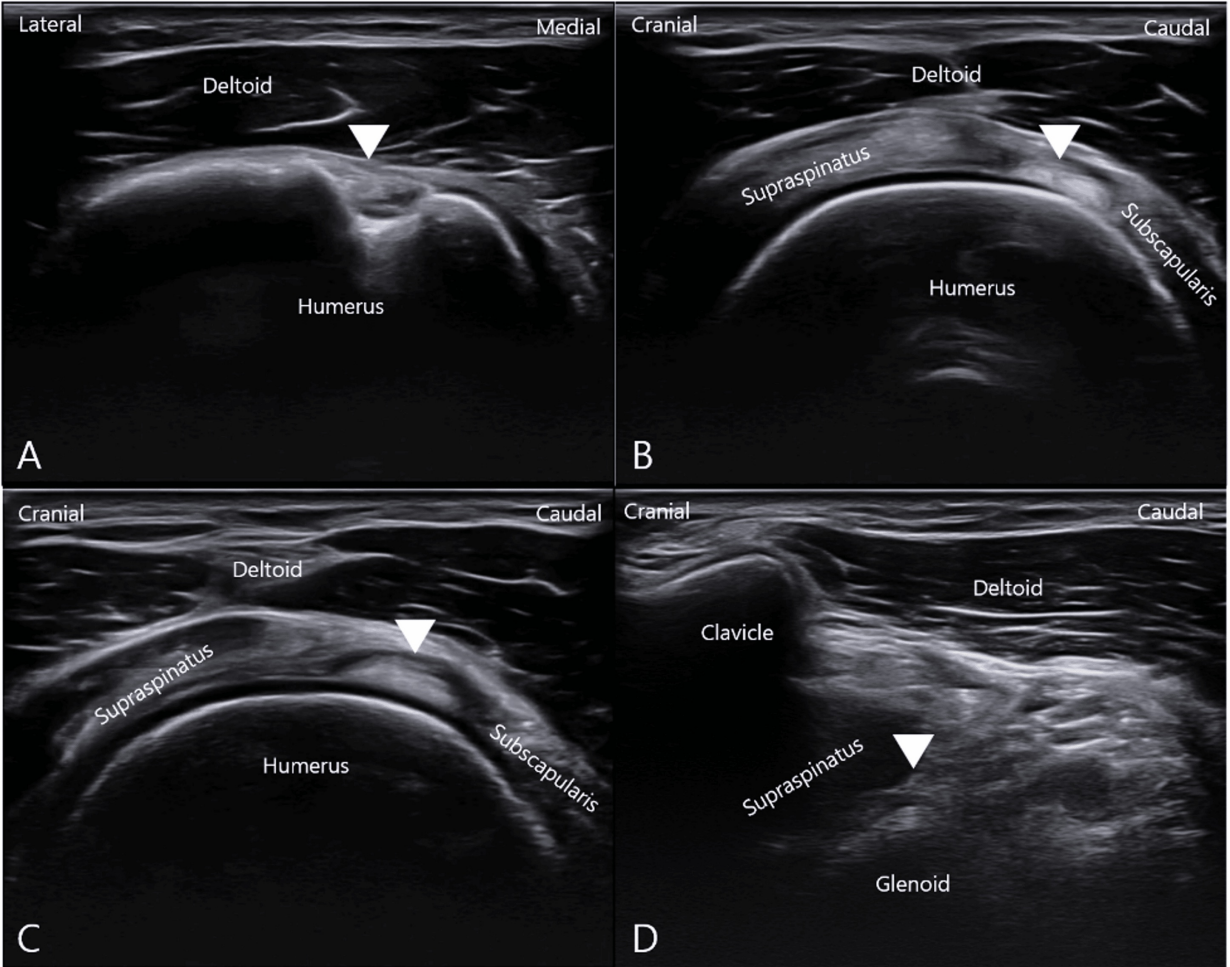
Stepwise short-axis ultrasound tracking of the long head of the biceps tendon to the superior labral-biceps anchor complex in a healthy volunteer Representative static short-axis ultrasound images of the structured biceps-tracking sequence. (A) LHBT is identified within the bicipital groove. (B) Proximal tracking through the rotator interval region. (C) Visualization of the LHBT entering its intra-articular course. (D) Final confirmation of the biceps anchor at the supraglenoid tubercle/superior labral attachment Image courtesy of Dr. Yonghyun Yoon

This stepwise sequence was used as a structured biceps-tracking strategy to guide needle trajectory planning. Representative static images of the tracking sequence are shown in Figure 2, and the dynamic distal-to-proximal tracking process is provided in Video 1. Representative sonographic images and videos were obtained in a healthy volunteer, whereas the injection and dissection findings were derived exclusively from the cadaveric specimen.

**Video 1:** Dynamic ultrasound scanning of the long head of the biceps, short-axis, distal-to-proximal Dynamic distal-to-proximal short-axis ultrasound tracking of the long head of the biceps tendon in a healthy volunteer, beginning at the bicipital groove and proceeding through the rotator interval to the biceps anchor at the supraglenoid tubercle/superior labral attachment

Because the superior labrum itself is only partially and indirectly visualizable on ultrasound, target confirmation did not rely on isolated direct visualization of labral tissue alone. Instead, it was based on a composite sonographic assessment, including: (1) continuous proximal tracking of the LHBT from the bicipital groove, (2) visualization of the tendon entering its intra-articular course, (3) localization of the apparent proximal attachment region near the supraglenoid tubercle/superior labral-biceps anchor complex, and (4) correlation with adjacent osseous landmarks and the constrained corridor between the coracoid process and clavicle.

Ultrasound-guided access and injectate delivery

Under real-time ultrasound guidance, a 23-gauge, 6-cm needle was advanced toward the superior labral-biceps anchor complex using an out-of-plane (OOP) technique. The needle was introduced from an approximately 3-cm lateral skin entry point relative to the transducer footprint and advanced in a lateral-to-medial direction at an insertion angle of approximately 45° relative to the skin surface. This lateral starting point was used as a procedural reference in this specimen and was not intended as a fixed universal measurement; it may require adjustment according to shoulder size, body habitus, transducer position, and the available acoustic corridor. The intended target depth was approximately 3 cm.

**Figure 3:**
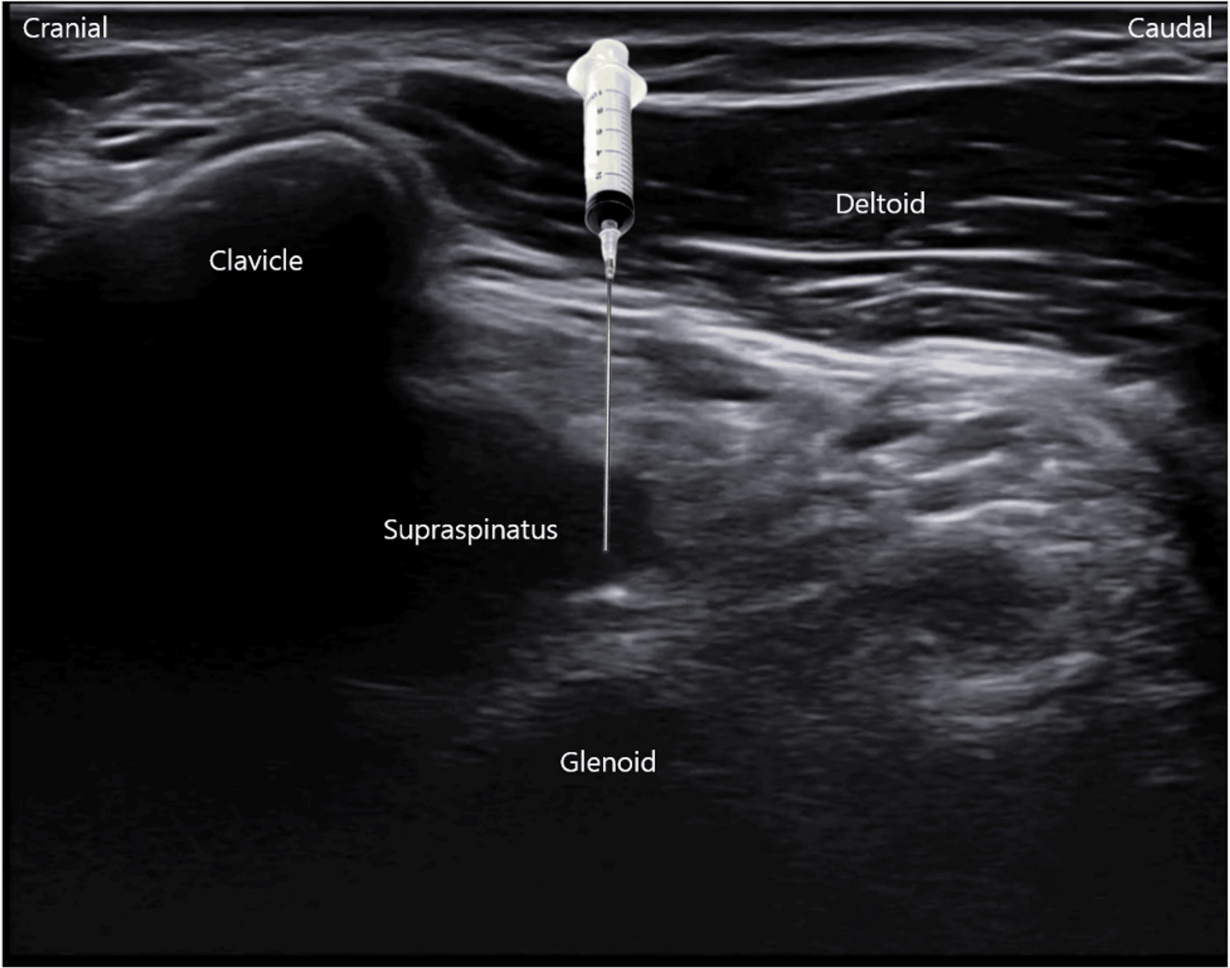
Ultrasound-guided out-of-plane needle approach for access to the superior labral-biceps anchor complex in the cadaveric specimen Representative short-axis procedural ultrasound image demonstrating the out-of-plane technique. The needle was advanced in a lateral-to-medial direction from a skin entry point approximately 3 cm lateral to the probe footprint, at an insertion angle of approximately 45°, toward a target depth of approximately 3 cm. Image courtesy of Dr. Yonghyun Yoon

Needle tip position during OOP advancement was confirmed using repeated trace-back scanning, with proximal-distal transducer sweeps along the needle shaft to repeatedly re-identify the tip relative to the target before injection. Because OOP visualization does not permit continuous full-length visualization of the shaft and tip in the same way as an in-plane approach, accurate tip localization depended on repeated dynamic rescanning and operator experience. A representative procedural ultrasound image is shown in Figure 3.

A notable technical constraint in both shoulders was the narrow acoustic and spatial window between the coracoid process and clavicle. This limited the available entry angle and required careful adjustment of both transducer position and needle angulation to maintain a trajectory toward the target without impingement on adjacent osseous structures. This corridor limitation was recorded qualitatively; no objective measurement of corridor width or allowable needle angulation was performed.

After target confirmation, 1 mL of injectate was delivered into each shoulder. The injectate consisted of a commercially available cosmetic hyaluronic acid-based filler mixed with red dye to facilitate gross identification during subsequent dissection. A relatively high-viscosity material was selected to reduce unintended spread beyond the intended footprint and to facilitate focal gross identification at dissection. The high-viscosity surrogate injectate may have favored focal retention and limited spread, thereby biasing the observed localization pattern. Needle placement and injectate delivery were monitored in real time throughout the procedure. The real-time injection sequence is provided in Video 2.

**Video 2:** Ultrasound-guided out-of-plane access to the superior labral-biceps anchor complex Real-time ultrasound-guided out-of-plane access to the superior labral-biceps anchor complex in the cadaveric specimen. The needle is advanced in a lateral-to-medial direction toward the biceps anchor region at approximately 45° to the skin surface.

Dissection and descriptive outcome assessment

Layer-by-layer anatomical dissection was performed by a professional anatomist with more than 10 years of cadaveric dissection experience, approximately one hour after injection. The dissection was carried out carefully to preserve and expose the superior labrum, biceps anchor, joint capsule, and adjacent rotator interval structures. The LHBT was identified and followed proximally to its origin at the supraglenoid tubercle, and the superior labrum-biceps anchor footprint was directly inspected.

The primary descriptive outcome, defined before dissection, was visible focal localization of red filler at the superior labral footprint, defined as the supraglenoid tubercle/biceps anchor region, without gross diffuse red-filler pooling within the glenohumeral joint cavity. Gross extension into the glenohumeral joint cavity or to regions posterior, medial, or anterior to the supraglenoid tubercle was also recorded descriptively. When present, the relationship between the red filler margin and the intended superior labral-biceps anchor footprint was assessed grossly with a ruler by the anatomist performing the dissection. This assessment was qualitative, approximate, and non-validated; no blinded assessment, interobserver reliability analysis, standardized grading system, volumetric analysis, histologic confirmation, fluoroscopic confirmation, magnetic resonance correlation, or quantitative three-dimensional spread mapping was performed.

After completion of the ultrasound-guided red-filler injection and before dissection, 10 mL of blue dye was injected into the glenohumeral joint cavity through an anterior intra-articular approach using a 23-gauge needle as part of a concurrent anatomical protocol. The purpose of the blue dye was to delineate the joint-cavity staining pattern and visually distinguish intra-articular blue staining from the red filler deposited at the intended superior labral-biceps anchor footprint. The blue dye injection was not used for ultrasound-guided target confirmation and was not intended to serve as a formal comparator arm.

Findings

Ultrasound-guided injections were performed in both shoulders of the single fresh-frozen specimen using the predefined protocol. Stepwise sonographic tracking of the LHBT from the bicipital groove to the supraglenoid tubercle was achieved in both shoulders. The target region was visualized bilaterally, although the narrow interval between the coracoid process and clavicle constrained the available needle trajectory in both procedures. A descriptive summary of the bilateral procedural findings is provided in Table 1.

**Table 1:** Descriptive summary of the bilateral procedural findings Summary of the two non-independent procedural attempts performed in the right and left shoulders of the single cadaver, including LHBT tracking, OOP access, major technical constraint, and gross injectate localization/spread findings. Findings are descriptive and based on gross anatomical evaluation only. LHBT, long head of the biceps tendon; OOP, out-of-plane.

Shoulder	LHBT tracking to target achieved	OOP access performed	Major technical constraint	Gross injectate localization	Diffuse intra-articular red pooling	Gross extension beyond the supraglenoid tubercle	Shoulder	LHBT tracking to target achieved	Shoulder	LHBT tracking to target achieved
Left	Yes	Yes	Narrow coracoid-clavicle corridor	Adjacent to the intended footprint on direct inspection	No	No	Left	Yes	Left	Yes
Right	Yes	Yes	Narrow coracoid-clavicle corridor	Adjacent to the intended footprint on direct inspection	No	No	Right	Yes	Right	Yes

A total of 1 mL of red-stained high-viscosity filler was delivered at the intended target in each shoulder. Real-time ultrasonography did not demonstrate grossly apparent needle deviation or visually evident unintended injectate spread during injection; however, sonographic appearance alone could not confirm exact footprint-level placement.

**Figure 4:**
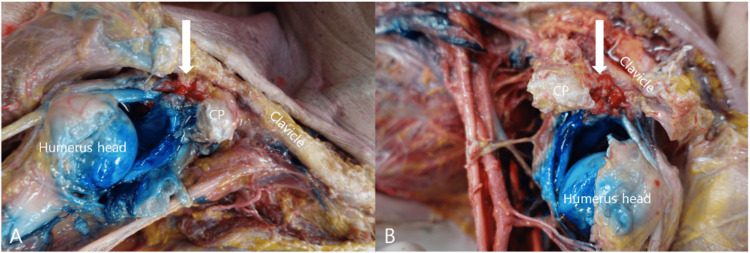
Cadaveric dissection demonstrating red filler localization at the superior labral-biceps anchor footprint Representative gross dissection photographs obtained after ultrasound-guided red-filler injection and subsequent intra-articular blue dye injection. White arrows indicate focal red filler localization at the long head of the biceps anchor on the supraglenoid tubercle. Blue staining represents glenohumeral joint-cavity dye that was injected after completion of the ultrasound-guided red-filler procedure and before dissection as part of a concurrent anatomical protocol. The blue dye was used to delineate the joint-cavity staining pattern and was visually distinguishable from the red high-viscosity filler. (A) Left shoulder, with the acromioclavicular joint preserved. (B) Right shoulder, with the acromioclavicular joint disarticulated to facilitate exposure. In both shoulders, focal red-filler deposition was identified at the intended footprint without diffuse red-filler pooling within the glenohumeral joint cavity. Image courtesy of Dr. Yonghyun Yoon

Layer-by-layer dissection performed approximately one hour later demonstrated focal red filler localization at the long head of the biceps anchor on the supraglenoid tubercle in both shoulders, corresponding to the intended superior labral footprint. In both shoulders, there was gross injectate localization adjacent to the intended footprint on direct inspection. No diffuse red-filler pooling was observed within the glenohumeral joint cavity, and no gross posterior, medial, or anterior extension beyond the supraglenoid tubercle was noted. Representative gross dissection findings are shown in Figure 4.

On the right side, the acromioclavicular joint was disarticulated to facilitate exposure of the superior labral region. In both shoulders, the localization pattern was descriptively consistent with focal footprint-level deposition rather than non-specific intra-articular pooling. Because this report involved a single donor and only two non-independent procedural attempts, procedural success rates cannot be estimated from the present data.

## Discussion

This technical report describes a structured ultrasound-guided biceps-tracking approach to the superior labral-biceps anchor complex and provides a cadaveric demonstration of gross injectate localization at the intended footprint. In both shoulders of a single fresh-frozen specimen, gross dissection demonstrated focal red-filler deposition at the supraglenoid tubercle/biceps anchor region without diffuse intra-articular pooling. These findings suggest preliminary anatomical feasibility of ultrasound-guided footprint-level access to this region in this cadaveric specimen.

Interpretation of the focal localization finding should be cautious because it may have been influenced by the physical properties of the injectate. The material used in this report was a red-stained cosmetic hyaluronic acid-based filler rather than a clinically intended therapeutic agent. Its relatively high viscosity may have favored focal retention and limited spread, thereby biasing the observed localization pattern. Accordingly, the present findings demonstrate anatomical localization using a high-viscosity surrogate marker injectate, but they do not establish that similar localization would be achieved with lower-viscosity materials commonly used in clinical practice.

The key technical feature of the approach was systematic proximal tracking of the LHBT from the bicipital groove to its origin. Rather than attempting to target the superior labral region directly from the outset, the technique used the anatomical continuity of the tendon as a sonographic guide. The rotator interval served as an intermediate corridor during this tracking process, providing progressive confirmation of tendon orientation toward the biceps anchor [25-28]. From a procedural standpoint, this stepwise sequence may provide an anatomical framework for approaching a region that is otherwise difficult to visualize sonographically.

A second procedural observation was the importance of the limited acoustic corridor between the coracoid process and the clavicle. In both shoulders, this narrow window constrained the available entry angle and influenced the needle trajectory. Under these conditions, an OOP lateral-to-medial approach was anatomically feasible, although it required repeated dynamic tip confirmation by trace-back scanning. Because only OOP access was tested, no conclusions can be drawn regarding whether OOP or in-plane access is preferable for this target. A direct comparative study would be required [29-32].

The present findings should not be interpreted as evidence that footprint-directed injection is clinically superior to conventional glenohumeral intra-articular injection. Rather, the report addresses a narrower anatomical question: whether the superior labral-biceps anchor footprint can be approached and grossly localized under ultrasound guidance using a structured biceps-tracking strategy. Any clinical benefit of footprint-directed targeting over standard glenohumeral intra-articular injection remains hypothetical. Potential methodological or clinical implications of footprint-level targeting are therefore speculative and hypothesis-generating only.

The distinction between footprint-level localization and joint-cavity injection is anatomically interesting but remains unproven as a clinically meaningful difference. In the present specimen, intra-articular blue dye produced a staining pattern that was visually distinguishable from the focal red-filler localization at the biceps anchor. Because this blue dye injection was qualitative and not designed as a controlled comparator, it cannot determine whether conventional intra-articular injection would or would not deliver clinically meaningful concentrations of injectate to the SLAP region. It does, however, provide a visual anatomical rationale for future controlled comparisons with standard glenohumeral injection techniques [17-24].

Although the present work is conceptually related to our prior cadaveric study of ultrasound-guided targeted access to the anterior labral-ligamentous complex, the anatomical target and procedural constraints are distinct [33]. The previous study focused on an anterior capsulolabral attachment region, whereas the present technique addresses the superior labral-biceps anchor complex at the supraglenoid tubercle. The contribution of the current report should therefore be understood as anatomical and technical rather than clinical: it extends feasibility work to a different capsulolabral attachment site with a different sonographic pathway and needle corridor.

Operator experience is also important in interpreting the findings. All procedures were performed by a single experienced musculoskeletal ultrasonography operator, and OOP needle guidance is inherently operator dependent. Reproducibility in less experienced hands is unknown, and inter-operator variation was not assessed. The positioning strategy used in this cadaver, including approximately 90° of external rotation, was selected to improve anterior exposure of the LHBT and rotator interval; however, the tolerance and reproducibility of this position in symptomatic living patients remain uncertain, particularly in the presence of pain, guarding, instability, or stiffness.

Limitations

This report has several important limitations. First, it involved a single fresh-frozen cadaver with bilateral injections from the same donor; therefore, the effective biological sample size was one, and the two observations were not statistically independent. Second, outcome assessment was based on gross dissection and approximate ruler-assisted inspection without volumetric mapping, fluoroscopic confirmation, magnetic resonance correlation, histologic confirmation, blinded assessment, or quantitative three-dimensional spread analysis. Third, no formal comparator condition was included, such as conventional ultrasound-guided glenohumeral intra-articular injection, an alternative needle trajectory, or an in-plane approach. Fourth, the injectate was a non-clinical, high-viscosity surrogate whose dispersion characteristics may differ substantially from those of clinically used injectates and may have favored focal retention at the target region. Fifth, all procedures were performed by a single experienced operator, so inter-operator reproducibility was not assessed. Sixth, because of the single-donor descriptive design, procedural success rates cannot be estimated from the present study.

Taken together, these limitations mean that the findings should be interpreted strictly as descriptive anatomical observations. They do not establish reproducibility, safety, comparative accuracy, or clinical efficacy.

## Conclusions

This technical report describes an ultrasound-guided biceps-tracking approach to the superior labral-biceps anchor complex with cadaveric demonstration of gross injectate localization at the supraglenoid tubercle/biceps anchor region. In this single-cadaver study, the technique appeared anatomically feasible but was constrained by a narrow acoustic corridor between the coracoid process and the clavicle. Localization was assessed by gross anatomical evaluation only, without quantitative imaging, volumetric analysis, or independent confirmation methods.

These findings are descriptive only and do not establish reproducibility, safety, comparative accuracy, procedural success rates, or clinical efficacy. Any clinical benefit over standard glenohumeral intra-articular injection remains hypothetical. Further cadaveric and clinical studies are needed to evaluate multi-specimen reproducibility, compare this approach with conventional glenohumeral intra-articular injection and alternative needle trajectories, assess multiple operators, and test clinically relevant injectates with different dispersion characteristics.
